# Historical dataset of administrative units with social-economic attributes for Austrian Silesia 1837–1910

**DOI:** 10.1038/s41597-020-0546-z

**Published:** 2020-06-30

**Authors:** Krzysztof Ostafin, Dominik Kaim, Tadeusz Siwek, Anna Miklar

**Affiliations:** 1 Jagiellonian University, Faculty of Geography and Geology, Institute of Geography and Spatial Management, Kraków, Poland; 2 University of Ostrava, Faculty of Science, Department of Human Geography and Regional Development, Ostrava, Czech Republic; 3 Jagiellonian University, Faculty of History, Institute of History, Kraków, Poland

**Keywords:** Geography, History

## Abstract

Scientists from many disciplines need historical administrative boundaries in order to analyse socio-economic data in space and time. In this paper, we present a set of historical data consisting of administrative unit boundaries and exemplary socio-economic attributes for Austrian Silesia, an historical region located in modern Czechia and Poland. The dataset covers nearly 700 administrative unit boundaries on the level of cadastral or political communes and their subparts and was acquired through manual vectorisation of historical maps (1:28,800) from the period 1837–1841. The local-level units can be easily joined into higher-level divisions such as court or political districts for the period 1837–1910. The data can then be combined with statistical data collected approximately every 10 years for a similar period. Within the quality assessment, the relations between cartographic and census data and their credibility are analysed. The present dataset provides many possibilities for joining a wide range of historical statistical data to better understand various demographic and economic processes based on advanced analyses, e.g., by using GIS.

## Abstract (summary)

**Table:** 

Measurement(s)	administrative region • Socioeconomic Indicator
Technology Type(s)	digital curation
Factor Type(s)	time period
Sample Characteristic - Environment	anthropogenic environment
Sample Characteristic - Location	Poland • Czech Republic

Machine-accessible metadata file describing the reported data: 10.6084/m9.figshare.12464801

## Background & Summary

With progress in the digitalisation of historical documents in libraries and archives e.g., Österreichischen Nationalbibliothek (www.onb.ac.at), Zemský archiv v Opavě (http://www.archives.cz), the availability of such documents to internet users increases. Although large collections of historical map scans and census and industrial data records, especially from the 19^th^ and beginning of the 20^th^ century, have been made available, the main barrier for their use is still the time- and resource-consuming process of data pre-processing (e.g., georeferencing, vectorisation, optical character recognition)^1^. Another major barrier in the inclusion of historical data in current analyses is administrative boundary changes in the regions about which data were collected. To make past data comparable with current data, detailed historical administrative boundary reconstruction is needed, preferably across different time periods^2^.

Incorporating the former extent of historical administrative units is critical for organising statistical data both spatially and temporarily in order to analyse and visualise it. There is a high demand for such data because it allows the extension of time series to the past and thus improves the analytical possibilities for understanding the consequences of historical, demographic and economic processes^3,4^.

Recently, several historical datasets at various spatial scales, ranging from individual units up to whole countries or regions, have been made available^5–7^. Sometimes, the data are also available in the form of geoportals with different levels of interactivity such as A vision of Britain trough time (www.visionofbritain.org.uk), Social Explorer (www.socialexplorer.com), HGIS Germany (www.hgis-germany.de), Database of the Ukrainian residents born between 1650 and 1920 (www.pra.in.ua), but most often, the acquisition of this data is limited. The scope of spatial and historical studies is still insufficient, especially for Central and Eastern Europe, where interest in the digital humanities is dynamically increasing^8,9^ and new projects appear e.g. HistoGIS (https://histogis.acdh-dev.oeaw.ac.at) or Der Franziszeische Kataster (www.franziszeischerkataster.at). In this region, due to complicated historical trajectories, many of the regions characterised by historical census data no longer exist, have changed their boundaries or were divided by political borders that have since come into existence. Therefore, many analyses are limited to the currently existing administrative divisions^10^. If proper and detailed historical administrative boundaries are available, the density of data time series and a detailed spatial scale could be substantially improved for many studies. For instance, the Czech Statistical Office (CZSO) shares its list of municipalities and villages along with the number of inhabitants and houses back to 1869^11^; however, the list lacks historical administrative boundaries. The Polish Local Data Bank (https://bdl.stat.gov.pl) has made statistical data only from 1995 on available.

Polish historians work on and share their demographic and economic data for southern Poland and western Ukraine (the historical region of Galicia) mainly as a generalised version of their original data sources^12,13^. These authors emphasise that changes in administrative boundaries are the main problem in data comparisons over time. Scholars conducting long-term analyses of local administrative units on the level of municipalities or single villages are few, and they have not shared their data with other scholars^14^.

**Fig. 1:**
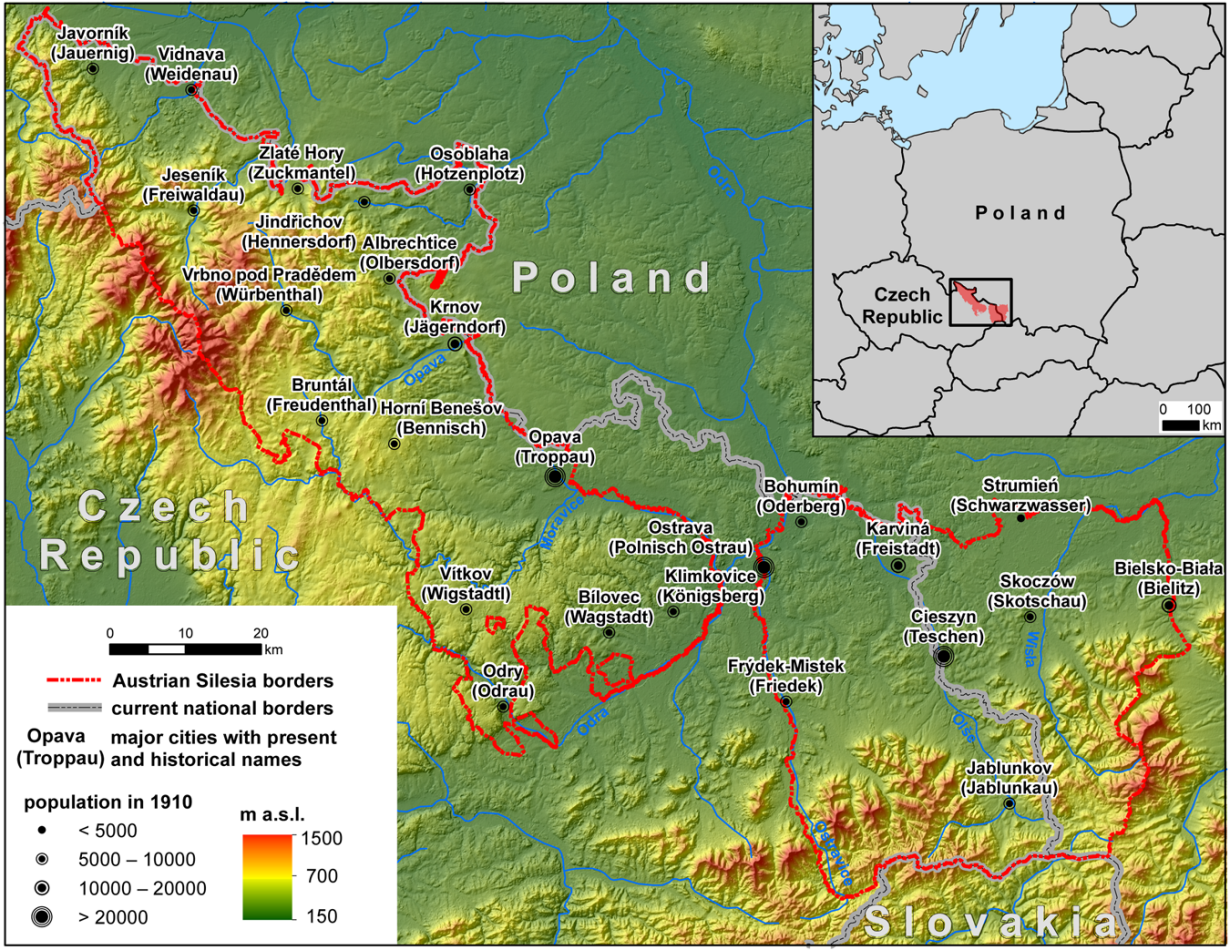
Historical boundaries of Austrian Silesia and current national borders.

Hence, the idea arose of developing and providing a set of data with a detailed administrative division and exemplary demographic and economic data for the historical region of Austrian Silesia (ger. *Österreichisch Schlesien*, cze. *Rakouské Slezsko*, pol. *Śląsk Austriacki*). This region consisted of two parts, Opavian Silesia (2864.7 km^2^, 55.6%) in the west and Cieszyn Silesia in the east (2285.8 km^2^, 44.4%), separated by a narrow wedge of Moravia from the south and Prussian Silesia from the north (Fig. 1). Austrian Silesia was formed after the Austrian-Prussian wars of Silesia (Silesian Wars) in the middle of the 18^th^ century, and until 1918, it was a province of the Austrian monarchy (from 1867, Austro-Hungarian). The region was influenced by the Czech Přemyslid dynasty, Polish Piast dynasty and Austrian Habsburg dynasty, as well as several nations and cultures^15,16^. It is also a very interesting area in terms of physio-geography, covering the eastern part of the Sudety Mountains and the western part of the Carpathians. At present, 80.4% of the research area is in the Czech Republic, including 55.9% of Cieszyn Silesia and 99.9% of Opavian Silesia. The remaining 44.1% of the territory of Cieszyn Silesia and 0.1% of Opavian Silesia are in Poland.

The geometry of the boundaries presented in the dataset is based mainly on the information from detailed historical topographic maps. A total of 700 administrative units on the level of single villages, towns, or even their parts were reconstructed. The boundaries are accompanied by a list of socio-economic attributes collected every 10 years. They contain names in three languages (Czech, German, Polish) and data on the number of inhabitants, the gender of inhabitants, the languages spoken by inhabitants, confessions, the number of buildings, the number of farm animals and land use. The user can easily attach additional data to the administrative boundaries, being assured of their correctness for a specified period. The methods proposed in the paper can be easily transformed to any of the regions of the Austro-Hungarian Empire because these kind of data are available for other regions in the Austrian (Austro-Hungarian) Monarchy e.g. on Mapire Portal (https://mapire.eu) or on Österreichischen Nationalbibliothek websites (www.onb.ac.at). Combined detailed data from historical maps and censuses may help in better understanding of socio-economic processes such as demographic changes^15,17–19^ or land use changes^20–23^, and their consequences over large areas of Europe.

## Methods

**Fig. 2:**
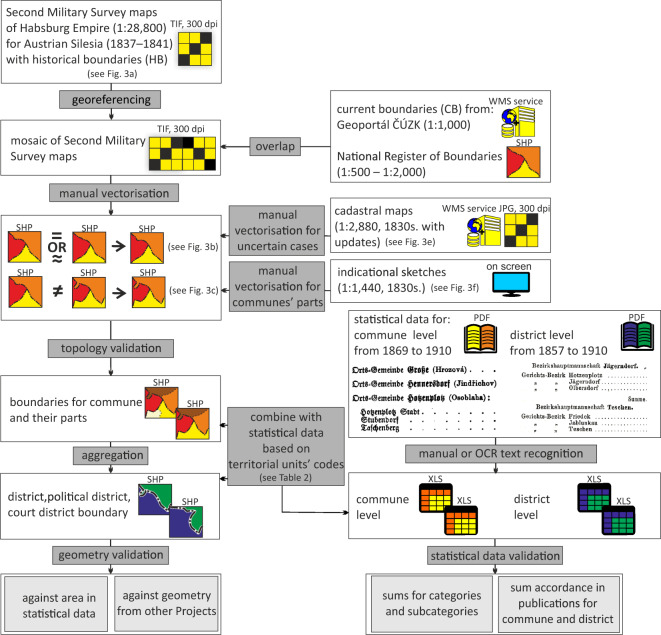
Workflow for the production of the dataset.

Thanks to the development of cartography and census data collection methods in the 19^th^ century, a large number of high-quality data were collected for Austrian Silesia. Currently, it is possible to join that data to contemporary datasets to extend the temporal series and to conduct long-term analyses by using new tools and formats, including GIS (Fig. 2).

**Fig. 3:**
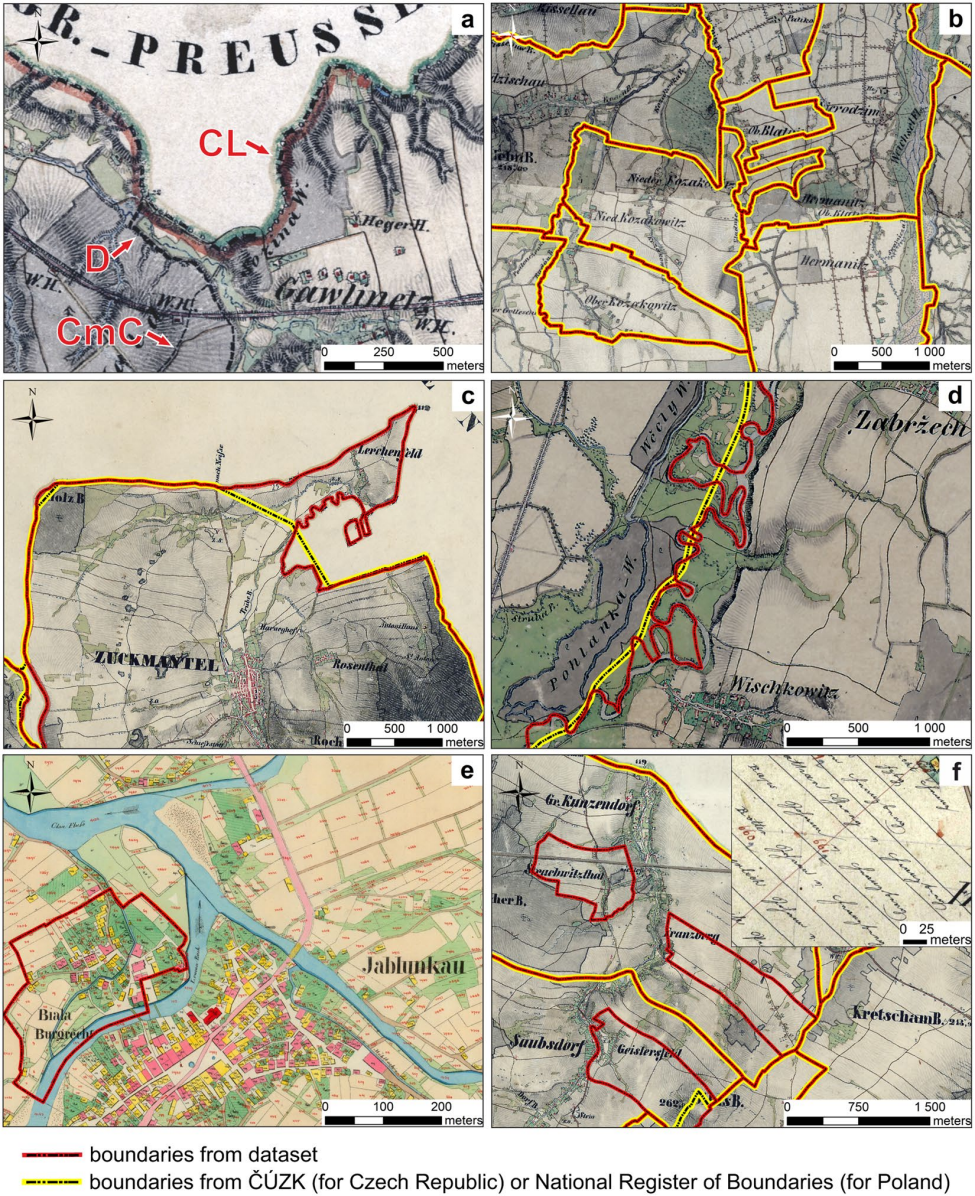
Examples of administrative boundary vectorisation based on historical maps. (**a**) Second military survey map (1:28,800) with boundaries: CL – Crown lands, D – districts, CmC – cadastral commune; (**b**) boundaries with no change; (**c**) change connected to the national boundary regulation; (**d**) change as a result of river regulation; (**e**) small administrative units presented on the cadastral mapping (1:2,880); (**f**) part of the cadastral/political commune on the second military survey with additional portion of indicational sketch (1:1,440) with the information on the origins of the owners (upper-right corner).

Administrative boundary reconstruction was based on manual vectorisation, as due to the diverse quality of the original maps, automatic image processing was very difficult. Although manual vectorisation is time-consuming, it may give better results with historical materials^24^. Usually, manual vectorisation serves as the reference for automatic procedures^25^. To obtain text data, optical character recognition (OCR) and manual methods were used. The advantage of the OCR is fast processing, but the disadvantage is the necessity of verification and corrections, especially with small and blurred font shapes or handwriting (Fig. 3).

### Cartographic data sources

As a basic cartographic material, 42 map sheets of the second military survey were used (1837–1841; 1:28,800). The maps are a generalisation of cadastral mapping (1:2,880) from the 1830s, updated with terrain relief information. The Austrian cadastre was founded by Emperor Franz in 1817. It served as a basis of stable spatio-temporal units (ger. *Stabiler Kataster*) for land taxation^26^. The maps were obtained from the War Archive in Vienna in the form of 300 dpi TIFF scans. As auxiliary data, cadastral maps (1:2,880) and indicative sketches (1:1,440) from the 1830s, which are available online in a digital form Český úřad zeměměřický a katastrální (ČÚZK) (https://archivnimapy.cuzk.cz/uazk/pohledy/archiv.html) and from Szukaj w Archiwach (www.szukajwarchiwach.gov.pl), were used. It was especially useful in situations where the quality of the topographic maps was very low and hard to interpret.

### Statistical data sources

In the 19^th^ and the beginning of the 20^th^ century, the administrative structure of Austrian Silesia changed several times. An important trigger of the reorganisation of the administrative system was the Spring of the Nations in 1848, with many socio-economic processes following^16^.

The basic administrative units for which the statistical data were collected were villages, towns and, in some cases, their parts (ger. *Orts, Ortschaft*), e.g., colonies or suburbs. The above-mentioned units, alone or in aggregation with other similar entities, created cadastral communes (ger. *Katastralgemeinden*) and political communes (ger. *Ortsgemeinden*). The cadastral commune was a unit created for tax collection purposes, while political communes were designed as the lowest level administrative and self-government units. In the case of a few towns, such as Troppau, Teschen, Freistadt, only part of the political commune belonged to the cadastral commune. In the Austrian Silesia cadastral commune covered only one political commune, usually without its parts. The boundaries of both were exactly the same. In 1900 in Austrian Silesia there were 484 cases. 100 cases of cadastral commune covered one political commune with parts thereof. Only in one case cadastral commune covered two political communes. The names of cadastral and political communes generally coincided, though with a few exceptions (e.g., Gurschdorf, Tierlitzko). Political communes, as autonomous units, obtained legal status after the abolition of serfdom in 1848. Earlier, rural political communes were subject to the rule of masters in dominions (lat. *dominium*). Over a dozen cadastral communes formed a tax district (ger. *Steuer Bezirke*), while political communes formed the court districts (ger. *Gerichts Bezirke*). One or more court districts formed political districts (ger. *Politische Bezirke*) with their own authorities (ger. *Bezirkhauptman*). Apart from the above-mentioned structure, three towns in the region had their own statutes (ger. *Städte mit eigenem Statut*): Troppau, Bielitz, and Friedek. Political and court districts, as administrative units, were created in the place of former districts (ger. *Kreis*) that were dissolved in 1850.

The selection of the time series available in the paper was dependent on the availability of the historical data. In 1857, the first census under the Austrian Monarchy was conducted, not only for military purposes^27^. The next editions were carried out approximately every 10 years until 1910. The full results of the census were published both on the political and court district level^28–30^, with some data categories also available for separate towns, villages or parts thereof ^31–36^. Statistical publications for towns, villages and their parts differed with the number of separate spatial units and the thematic coverage in different periods. For instance, for the town of Wisla, data from 1850, 1869, 1880, 1900, and 1910 were available for two units, while for 1890, the data were available for 33 units within the town. Statistical sources do not have a uniform thematic scope. In most cases, the number of data collected increased with every next census, e.g., the number of cattle-related categories increased from 13 in 1857 to 40 in 1910. In some censuses, the definitions of categories were changed. However, in most cases, the sub-categories can be easily joined into more general categories, e.g., the numbers of inhabited and uninhabited buildings into the number of buildings or the number of Roman, Greek and Armenian Catholics into the number of Catholics. The published number of sub-categories and categories is larger for districts than for communes or their parts. For instance, the number of houses for communes in 1880 is summarised, while for the districts, it is presented separately for inhabited, uninhabited and parts of homes.

### Georeferencing

All of the second military map sheets were georeferenced by first-order polynomial transformation in ArcMap by using contemporary World Imagery satellite images (https://www.arcgis.com/home/item.html?id=10df2279f9684e4a9f6a7f08febac2a9) as a source of control points. Additionally, the official administrative unit’s data from the Czech Geoportal (ČÚZK) (https://geoportal.cuzk.cz) and National Register of Boundaries for Poland (www.gugik.gov.pl/pzgik) were used to collect the proper points. In most cases, the locations of characteristic road crossings, buildings or administrative boundary crossings were used as the control points. Altogether, 1083 points were used, which resulted in RMS values between 12 m and 21 m for 75% of the map sheets. The minimal RMS value was 6.6 m, and the maximal was 27.4 m. These results are comparable to other georeference works done for the second military survey in other parts of the Habsburg Empire^37^.

### Data acquisition

The manual vectorisation was conducted with a scale of 1:2000 – 1:5000 (Fig. 3a). During the acquisition, the tools of the ArcMap 10.7 Editor were used. The generalisation of the administrative boundaries on the second military survey versus cadastral mapping was limited. However, distinguishing the boundaries from other line symbols, such as roads, relief or densely settled areas, is not always easy. For both territories of contemporary Czechia and Poland, the use of the current administrative borders from ČÚZK (https://geoportal.cuzk.cz) and the National Register of Boundaries for Poland (www.gugik.gov.pl/pzgik) was very useful. If the current and historical boundaries overlapped, then the manual vectorisation followed the current data (Fig. 3b). If there were some discrepancies between the historical and current boundaries, a detailed inspection of the situation influenced the final decision (e.g., if the difference depended on real change, RMS error, or map distortion). Numerous boundary changes occurred along the historical boundary between the Habsburg Empire and Prussia (Fig. 3c). Currently, it is the Polish-Czech national boundary, with small corrections made in the 1950s. During the reconstructions, historical changes made before the river regulations (Fig. 3d) and water reservoir creations and small, local changes were considered. For several cadastral communes, the boundaries were acquired directly from cadastral mapping (1:2,880) (Fig. 3e). Finally, for 1900, the statistics cover 585 cadastral and 495 political communes^35^.

Additionally, thanks to the indicational sketches and analysis of the origin of the owners of the plots, it was possible to separate parts of communes (e.g., colonies, settlements), which resulted in increasing the spatial resolution of the dataset (Fig. 3f). In this way, for instance, in the Freiwaldau District, the number of basic administrative units increased from 58 cadastral communes to 108 units for 1900. Finally, nearly 700 administrative unit boundaries were reconstructed. In each case, the area of the vectorised units was verified by the statistical data. If statistical publications did not contain socioeconomic data for some part of commune, even though we had their geometry, those parts of commune were combined into larger administrative units, i.e. political or cadastral commune in accordance with generalised data in the statistical publication. However, this was the case in less than 5% of the units for each of the time periods.

Statistical data used in the study were acquired manually or semi-automatically by OCR ABBYY Fine Reader 12 software. For the oldest dataset, only the font shapes and signs unique to Czech, Polish or German were problematic. In some sources, single records were difficult to interpret due to the quality of the materials (e.g., distinguishing between 1 and 4 in some cases).

## Data Records

**Fig. 4:**
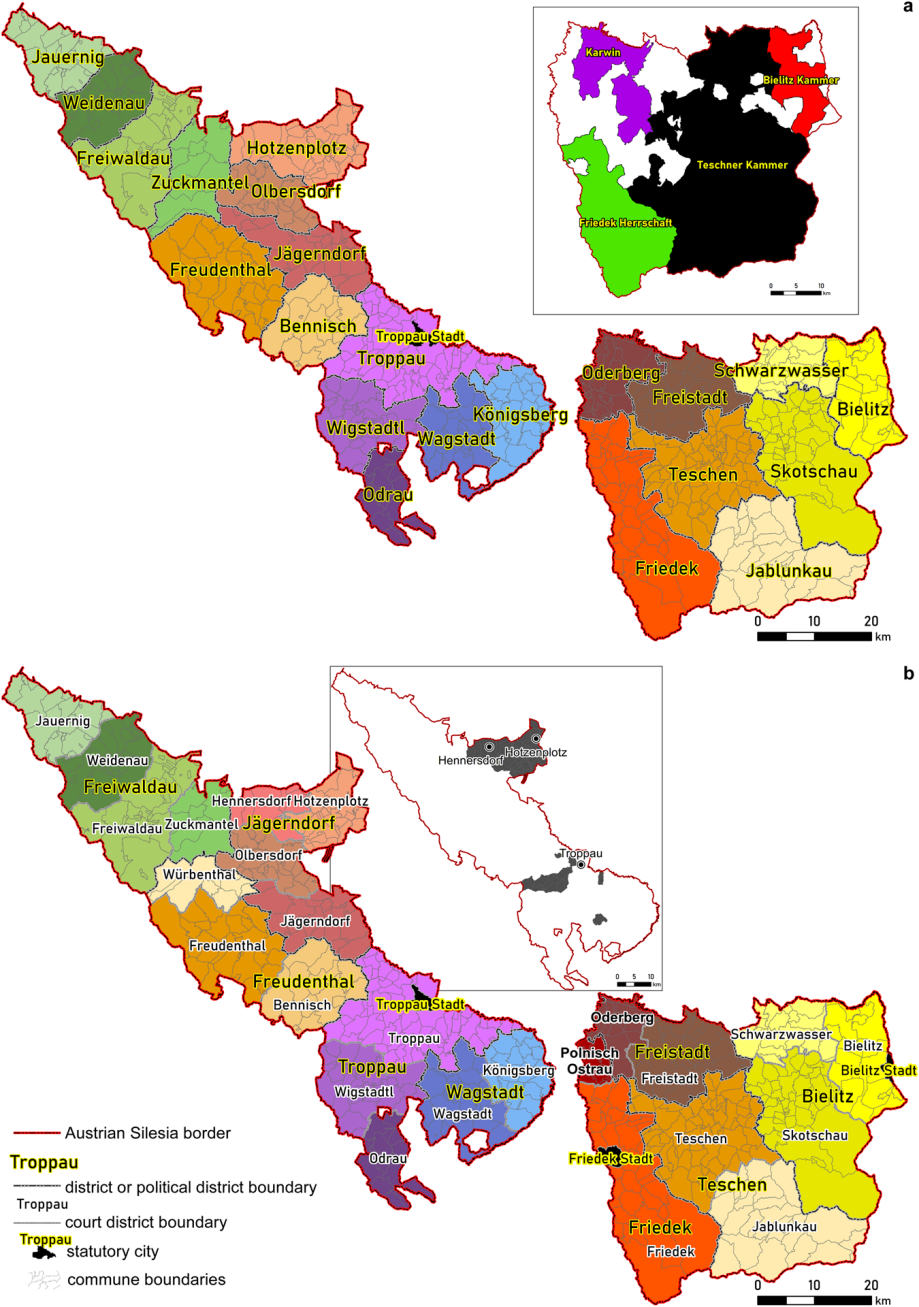
Districts of Austrian Silesia for 1857 (**a**) and 1910 (**b**). The additional maps present the main dominions in Cieszyn Silesia and Moravian enclaves on Oppava Silesia. Full list of administrative divisions available in Supplementary Information (Supplementary File 1).

The dataset^38^ presented in the paper is an open, vector SHP file that is easy to use in most GIS software and easily convertible to other formats. The shared layers are available on the level of communes and their parts and for districts for seven time periods between the 1830s and 1910 (Supplementary File 1). Altogether, 21 layers are available (Table 1). The names of the layers consist of two letters (SA, denoting Austrian Silesia), the years of the time period and spatial reference units: political communes (CmP), cadastral communes (CmC), court districts (DC) and political districts (DP). Communes can be easily combined into more complex units incl. older administrative units (e.g., dominions before 1848) or districts (Fig. 4), using simple dissolve- or selection-type functions. For instance, the territorial units codes for court district, political district and statutory town each consist of six numbers separated by slashes, e.g., 01/01/01, and for dominions, the maximum length of the code is 21 numbers separated by slashes (Table 2).

**Table 1:** List of administrative units covered by the dataset.

Layer	Dataset name	Geometry	Year
Political communes and parts thereof	SA_YYYY_CmP.shp	polygon	1837–1857; 1869; 1880; 1890; 1900; 1910
Cadastral communes and parts thereof	SA_YYYY_CmC.shp	polygon	1837–1857; 1900
Court districts	SA_YYYY_DC.shp	polygon	1850; 1869; 1880; 1890; 1900; 1910
Political districts	SA_YYYY_DP.shp	polygon	1850; 1857; 1869; 1880; 1890; 1900; 1910

**Table 2:** Codes of the territorial units.

Code	Level units	Short
01	Crow Land (ger. Land)	CL
02	Circle (ger. Kreis)	Cr
03	District, political district, statutory cities (ger. Bezirk, Land Bezirk, Politische Bezirk, Städte mit eigenem Statut)	DP
04	Court or judicial district (ger. Gerichtsbezirk)	DC
05	Tax district (ger. Steuerbezirk)	DT
006	Political commune (ger. Ortsgemeinde)	CmP
1	Part of political commune (ger. Ortschaft)	CmPp
007	Cadastral commune (ger. Katastralgemeinde)	CmC
1	Part of cadastral commune (ger. Katastralgemeinde)	CmCp
000	Dominion (ger. Landgut, lat. Dominium)	d

The spatial data are available with exemplary attributes covering geographical names, the number of buildings, demographic data, land use and cattle statistics in order to show the possibilities for data integration. An important reason for choosing exemplary attribute data was the ability to verify their values based on independent data sources for communes or districts. The selected attributes presented here highlight the potential of the statistics collected by the Habsburg administration^27^.

The first part of the attribute name consists of five characters describing the category and sub-category. Four digits following the space sign ‘_’ define the time period (YY) and the data source level (XX).

In the dataset presented in the paper, 4 name-related (Table 3), 4 house-related (Table 4), 42 demographic-related (Table 5), 9 farm animal-related and 5 land use-related attributes (Table 6) were selected. Commune names are available for 5 time periods: the 1830s, based on a second military survey, 1850^31^, 1880^33^, 1900^35^ and today, according to the official national geoportals (https://geoportal.cuzk.cz; www.geoportal.gov.pl). The names are presented in one, two or three languages (Czech, German, Polish), according to the source information availability. The census from 1880 is the only one that contains the local names in all three languages for almost all the communes covered. Some names of the same units may differ over time, which might be a result of phonetic records or problems with the transliteration of Slavic languages into German. The various versions of the names, however, are preserved here for unambiguous identification with other historical sources. Demographic data for almost all time periods include the population divided by gender.

**Table 3:** The name-related attributes covered by the dataset.

Attributes	Years		Description
	For Communes	For Districts	
Code_YYYY	1850, 1869, 1880, 1890, 1900, 1910	1850, 1857, 1869, 1880, 1890, 1900, 1910	territorial unit code
Nger_YYXX	1837–1841, 1850, 1880, 1900	1850, 1857, 1880, 1900, 1910	German name
Ncze_YYXX	1850, 1880, 1900, 2019	1880, 1900, 1910	Czech name
Npl_YYXX	1880, 1900, 2019	1880, 1900, 1910	Polish name
Ngedo_YYXX	1850	unavailable	German name of old dominions
EncM_YYXX	1890, 1900, 1910	unavailable	Moravian enclaves on Silesia
Arha_YYXX	1850, 1869, 1880, 1890, 1900, 1910	1850, 1869, 1880, 1890, 1900, 1910	area in ha

**Table 4:** The house-related attributes covered by the dataset.

Attributes	Years		Description
	For Communes	For Districts	
Hs_YYXX	1869, 1880, 1890, 1900, 1910	1857, 1869, 1880, 1890, 1900, 1910	number of houses
Hsoc_YYXX	unavailable	1869, 1880, 1890, 1900, 1910	number of occupied houses
Hsuoc_YYXX	unavailable	1869, 1880, 1890, 1900, 1910	number of unoccupied houses
Hspt_YYXX	unavailable	1857, 1869, 1880, 1890, 1900, 1910	number of part of houses

**Table 5:** The demographic-related attributes covered by the dataset.

Attributes	Years		Description
	For Communes	For Districts	
P_YYXX	1869, 1880, 1890, 1900, 1910	1857, 1869, 1880, 1890, 1900, 1910	local population present
Pcv_YYXX	1880, 1890, 1900, 1910	1880, 1890, 1900, 1910	civil population present
Pmil_YYXX	1880, 1890, 1900, 1910	1880, 1890, 1900, 1910	military population present
Pm_YYXX	1869, 1880, 1890, 1900, 1910	1857, 1869, 1880, 1890, 1900, 1910	number of men present
Pwm_YYXX	1869, 1880, 1890, 1900, 1910	1857, 1869, 1880, 1890, 1900, 1910	number of women present
Pmsg_YYXX	unavailable	1857, 1869	number of unmarried men
Pwsg_YYXX	unavailable	1857, 1869	number of unmarried women
Pmma_YYXX	unavailable	1857, 1869	number of married men
Pwma_YYXX	unavailable	1857, 1869	number of married women
Pmdv_YYXX	unavailable	1869	number of divorced men
Pwdv_YYXX	unavailable	1869	number of divorced women
Pmwi_YYXX	unavailable	1857, 1869	number of widowers
Pwwi_YYXX	unavailable	1857, 1869	number of widows
Pstr_YYXX	1910	1857, 1910	number of foreigners
Pger_YYXX	1880, 1890, 1900, 1910	1880, 1890, 1900, 1910	number of native German-speaking people
Pcms_YYXX	1880, 1890, 1900, 1910	1880, 1890, 1900, 1910	number of native Czech, Slovak, Moravian-speaking people
Ppl_YYXX	1880, 1890, 1900, 1910	1880, 1890, 1900, 1910	number of native Polish-speaking people
Pru_YYXX	as other-speaking PothS_YYXX in: 1880, 1890, 1900, 1910	1880, 1890, 1900, 1910	number of native Rusyn-speaking people
Psl_YYXX		1880, 1890, 1900, 1910	number of native Slovenian-speaking people
Psc_YYXX		1880, 1890, 1900, 1910	number of native Serbo-Croatian-speaking people
Pld_YYXX		1880, 1890, 1900, 1910	number of native Ladin-speaking people
Pro_YYXX		1880, 1890, 1900, 1910	number of native Romanian-speaking people
Phu_YYXX		1880, 1890, 1900, 1910	number of native Hungarian-speaking people
Pcr_YYXX	as Catholics Pcat_YYXX in: 1880, 1890, 1900, 1910	1857, 1869, 1880, 1890, 1900, 1910	number of Roman Catholics
Pcgr_YYXX		1857, 1869, 1880, 1890, 1900, 1910	number of Greek Catholics
Pcar_YYXX		1857, 1869, 1880, 1890, 1900, 1910	number of Catholics using the Armenian Rite
Pevlu_YYXX	as Evangelical Pev_YYXX in: 1880, 1890, 1900, 1910	1857, 1869, 1880, 1890, 1900, 1910	number of Lutherans (Augsburg Confession)
Pevhl_YYXX		1857, 1869, 1880, 1890, 1900, 1910	number of Helvetic Protestants
Pisr_YYXX	1880, 1890, 1900, 1910	1857, 1869, 1880, 1890, 1900, 1910	number of Israelites
Pcalt_YYXX	as others PothR_YYXX in: 1890, 1900, 1910	1880, 1890, 1900, 1910	number of Old Catholic Church believers
Pnugr_YYXX		1857, 1869, 1880, 1890, 1900, 1910	number of non-Uniate Greek Church believers
Pnuar_YYXX		1857, 1869, 1880, 1890, 1900, 1910	number of non-Uniate Armenian Church believers
Pmrvn_YYXX		1890, 1900, 1910	number of Moravian Church believers
Pang_YYXX		1880, 1890, 1900, 1910	number of Anglicans
Pmnnt_YYXX		1880, 1890, 1900, 1910	number of Mennonites
Punt_YYXX		1857, 1869, 1880, 1890, 1900, 1910	number of Unitarians
Plpv_YYXX		1890, 1900, 1910	number of Lipovans
Pmhmd_YYXX		1880, 1890, 1900, 1910	number of Muslims
Pothr_YYXX		1857, 1869, 1880, 1890, 1900, 1910	number of other believers
Punbl_YYXX		1880, 1890, 1900, 1910	number of unbelievers
Pcoth_YYXX	unavailable	1869	number of other Christians
Pncot_YYXX	unavailable	1869	number of other non-Christians

**Table 6:** The farm animal-related and land use-related attributes covered by the dataset.

Attributes	Years		Description
	For Communes	For Districts	
Ahors_YYXX	1900	1857, 1869, 1880, 1900, 1910	number of horses
Acttl_YYXX	1900	1857, 1869, 1880, 1900, 1910	number of cattle
Ashp_YYXX	1900	1857, 1869, 1880, 1900, 1910	number of sheep
Apg_YYXX	1900	1857, 1869, 1880, 1900, 1910	number of pigs
Abe_YYXX	unavailable	1869, 1880	number of hives
Ahn_YYXX	unavailable	1900, 1910	number of hens
Ags_YYXX	unavailable	1900, 1910	number of geese
Adk_YYXX	unavailable	1900, 1910	number of ducks
Apoth_YYXX	unavailable	1900, 1910	number of other poultries
LUarl_YYXX	1900	unavailable	area of arable lands
LUmd_YYXX	1900	unavailable	area of meadows
LUpst_YYXX	1900	unavailable	area of pastures
LUorc_YYXX	1900	unavailable	area of orchards
LUfrs_YYXX	1900	unavailable	area of forests

## Technical Validation

### Spatial data

**Fig. 5:**
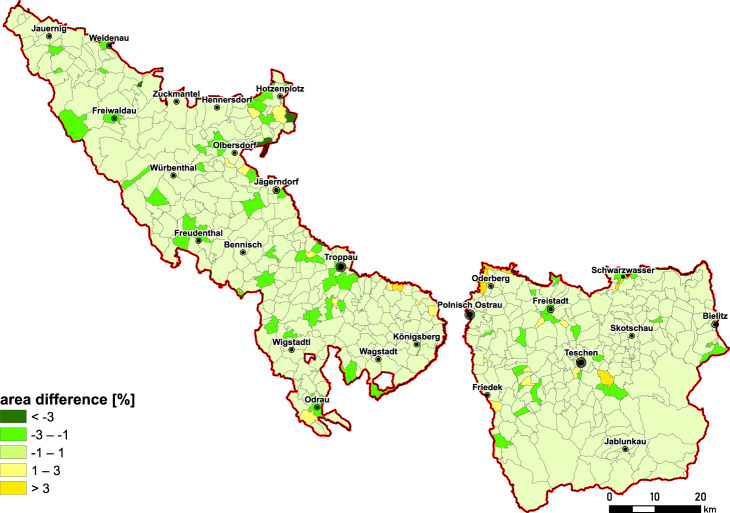
The comparison of area differences of cadastral communes (1837–1841) based on manual vectorisation and census data (1900).

The data acquired by manual vectorisation were inspected by topological correctness tools in ArcMap 10.7 by using the rules *Must Not Overlap* and *Must have no Gaps*, with a tolerance of 0.001 m. The area of cadastral communes can be verified with census data from 1900^35^. Other census data refer to the area of political communes or districts. For 91% of the cadastral communes, the differences between spatial and census data are between −1% and 1% and are between −3% and 3% (Fig. 5) for 97%. These results show the high quality of the source data and vectorisation procedure. Small discrepancies may be a result of the data processing in GIS (e.g., georeferencing) and/or slight changes in cadastral communes’ boundaries in the period under study (e.g., in Mährisch Pilgersdorf in 1846 or Kopitau in 1868).

The aggregation of communes into political districts confirms small (up to ±0.3%) discrepancies with census data (Table 7).

**Table 7:** Area comparison for districts based on vectorised cadastral communes’ aggregation and census data for 1857^28^ and 1910^36^.

District/Statutory city	Geometry area (ha)	Statistical area (ha)	Difference (%)
for 1857*			
Bielitz	76315	76364	−0.1
Freiwaldau	78760	78723	0.0
Jägerndorf	107250	107208	0.0
Teschen	152057	151978	0.1
Troppau	99356	99381	0.0
Troppau Stadt	1090	1093	−0.3
**for 1910**			
Bielitz	75818	75813	0.0
Freistadt	31766	31689	0.2
Freiwaldau	73667	73638	0.0
Freudenthal	59165	59165	0.0
Jägerndorf	53176	53220	−0.1
Teschen	73047	73038	0.0
Troppau	64231	64210	0.0
Wagstadt	35125	35144	−0.1
Friedek	46204	46171	0.1
Troppau Stadt	1090	1093	−0.3
Bielitz Stadt	497	497	0.0
Friedek Stadt	1022	1023	−0.1

Full coverage is available in the Supplementary Information (Supplementary File 1). *Area based on 1857 census data in Austrian miles converted to hectares.

**Fig. 6:**
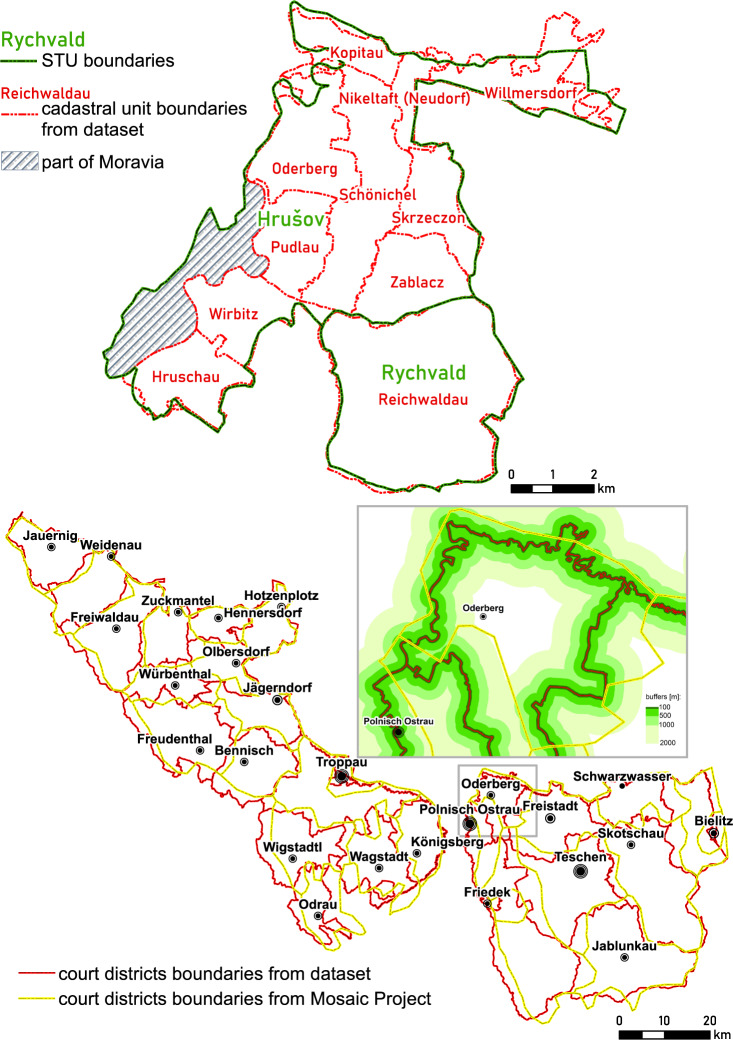
Comparison of the vectorised cadastral communes from the dataset with STUs (**a**) and court districts from the MOSAIC Project (**b**).

The boundaries vectorised manually were compared to the boundaries created by other authors. Bičik *et al*.^10,39^ created so-called STUs (stable territorial units) and BTUs (basic territorial units) consisting of 1 to 12 cadastral communes for the whole territory of Czechia, including Austrian Silesia (Fig. 6a). The units are stable over time for the period 1845–2010. According to this delineation, the number of cadastral communes in the Czech part of Austrian Silesia was generalised in nearly 40% of Austrian Silesia, from 473 cadastral communes in the 19^th^ century to 285 STUs. The average area of STUs for Austrian Silesia was 1590 ha, while for the cadastral commune, it was 874 ha. In some STUs, the Austrian Silesia communes were joined with those from Moravia or the Silesian part of Prussia. On the one hand, such aggregation allow the possibility of integrating data over large periods from various sources, but on the other hand, it lowers the spatial resolution of the data, which may negatively affect the ability to join such data with other historical or contemporary sources. For instance, the current availability of remote sensing data, which are independent from administrative units, makes it possible to compare land use directly to detailed historical boundaries.

The vectorised boundaries of cadastral communes aggregated to court districts were compared to the independent reconstruction prepared for the whole territory of the Habsburg Empire in 1910 during the MOSAIC Project (https://censusmosaic.demog.berkeley.edu) (Fig. 6b). This shapefile was created by Helmut Rumpler and Martin Seger^40^ and was slightly modified for the Max Planck Institute for Demographic Research and Chair for Geodesy and Geoinformatics, University of Rostock Population History GIS Collection. A total of 1711 court districts (incl. 28 for Austrian Silesia) were created during the project. The direct difference in district area between the MOSAIC Project and the dataset is 21.2%. For statuary towns, this difference is up to 80.9%, while for the rest of the districts, it is 14.1% (min – 0.5%, max – 54.4%). The generalisation of the boundary lengths between the two projects is equal to 22.4% (1862.4 km versus 2401.5 km). The boundary shifts of the MOSAIC-based geometry in the 100, 500, 1000 and 2000 m buffers around the dataset boundaries were calculated. Of the MOSAIC boundaries, 8.2% are located up to 100 m from dataset boundaries, while this figure is 35.8% in the 500 m buffer, 59.7% in the 1000 m buffer and 86.8% in the 2000 m buffer. This simple comparison shows the impact of the scale of the original cartographic sources used for the reconstruction on the final results.

### Census data

**Fig. 7:**
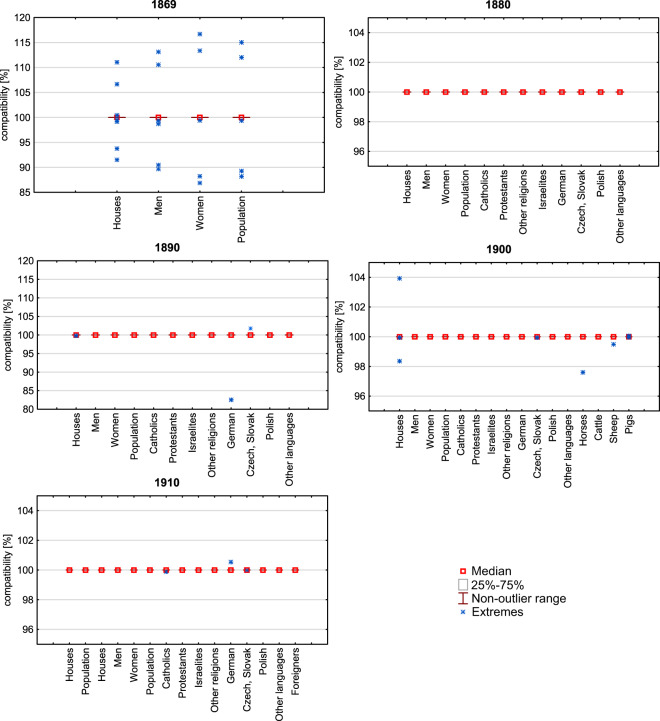
The sums were calculated for the communes aggregated to the district level and for the data published on the court district level. The analysis was based on 682 communes and their parts versus 24 court districts for 1869, 700 versus 27 for 1880, 685 versus 27 for 1890, 674 (CmP) or 585 (CmC) versus 27 for 1900 and 668 versus 28 for 1910.

The credibility of census data from Habsburg statistical institutions has been the subject of many studies confirming their high scientific value as a result of advanced data collection methods^27,41^. However, the quality of census publications is so far less recognised. In the dataset presented in this paper, the data available for communes were aggregated to the level of court districts for each of the time periods and compared to the sums available for the level of court districts 1) within the same source and 2) with the independent source (Fig. 7). The criterion of categories and sub-categories chosen was the availability of data for conducting a comparison on two administrative levels.

First, inconsistencies in the data may be verified by the partial, categorical and sub-categorical sums (e.g., the number of men and women should be summed to the category ‘population together’, and sums for exemplary category for court district level should be equal to the summed level of superior political district). Second, it needs to be verified by analysing partial sums which publication the error appeared in. Sum accordance in both publications confirms the credibility of both. The above-mentioned methods enabled the removal of errors triggered by the conversions of raster to text during the data acquisition process. The accordance expressed by the median, non-outlier range and the value of extremes confirm the rare errors in the source materials, regardless of the time period. Sums of exemplary categories and sub-categories aggregated to court districts are equal to the values published for court districts in almost 100% of the cases. For instance, for more than 680 political communes in 1869, there are only 3 errors in the records. On page 1, the data were exchanged for the communes of Alt Bielitz and Batzdorf, and on page 18, in the commune of Nider Hillersdorf, the total population exceeded the sum of men and women. For 1880 for all the categories published for the communes, no errors were detected when comparing the values to district level publications. For 1890, two errors were found: 7 for 1900 and 3 for 1910.

## Usage Notes

We shared the data in open, widely used shapefile format. According to shapefile format specification, boundary geometries are available in a direct access, main file with shp extension. In the shx index file, each record contains the offset of the corresponding main file record from the beginning of the main file. The socio-economic data, as dBASE table with one record per feature, are available in dbf extension. The one-to-one relationship between geometry and attributes is based on a record number. Attribute records in the dBASE file are the same order as records in the main file.

Examples of mandatory files for shapefile format:

- ***SA_1837_1850_CmC.shp***
- ***SA_1837_1850_CmC.shx***
- ***SA_1837_1850_CmC.dbf***

Examples of other files for shapefile format:

- ***SA_1837_1850_CmC.cpg*** — used to specify the code page (only for.dbf) for identifying the character encoding to be used
- ***SA_1837_1850_CmC.prj*** — projection description with text representation of coordinate reference systems
- ***SA_1837_1850_CmC.sbn*** and ***SA_1837_1850_CmC.sbx*** — a spatial index of the features
- ***SA_1837_1850_CmC.shp.xml*** — geospatial metadata in XML format

In the second version of the dataset each shapefile has its own metadata XML file, based on ISO 19139 Metadata Implementation Specification GML 3.2 standard, where the details such as sources of socio-economic data with pages or map sheets are clarified.

The shapefiles are stored in 21 compressed ZIP archives. The ZIP archives can be opened directly using QGIS open software https://qgis.org or after unpacking using e.g. 7-Zip https://www.7-zip.org/ or WinZip https://www.winzip.com also load with the single command Add Data to ArcGIS Desktop or ArcGIS Pro https://www.arcgis.com.

We ask the reader to refer to Tables from 1 to 6 for detailed explanations of the different data layers and fields.

## Supplementary Information

Supplementary Information

## References

[1] Jackson, M. A. & Woodsford, P. A. GIS data capture hardware and software. In *Geographical Information Systems: Priciples and Applications* (eds. Maguire, D. J., Goodchild, M. F. & Rhind, D.) 239–249 (Longman, 1991).

[2] Gregory, I. N. & Southall, H. R. Mapping British Population History. In *Past Time, Past Place GIS for History* (ed. Knowles, A. K.) 117–130 (ESRI Press, 2002).

[3] Munteanu, C. *et al*. Land Change in the Carpathian Region Before and After Major Institutional Changes. In *Land-Cover and Land-Use Changes in Eastern Europe after the Collapse of the Soviet Union in 1991* (eds. Gutman, G. & Radeloff, V.) 57–90, 10.1007/978-3-319-42638-9_4 (Springer, 2017).

[4] Gingrich S, Krausmann F (2018). At the core of the socio-ecological transition: Agroecosystem energy fluxes in Austria 1830–2010. Sci. Total Environ..

[5] Hedefalk F, Svensson P, Harrie L (2017). Spatiotemporal historical datasets at micro-level for geocoded individuals in five Swedish parishes, 1813–1914. Sci. Data.

[6] Leyk S, Uhl JH (2018). HISDAC-US, historical settlement data compilation for the conterminous United States over 200 years. Sci. Data.

[7] Lieskovsky J (2018). Historical land use dataset of the Carpathian region (1819–1980). J. Maps.

[8] Szady B (2013). Geografia historyczna w Polsce – rozwój i perspektywy. Stud. Geohistorica.

[9] Semotanová E, Chromý P (2012). Development and current trends of the Czech historical geography. Hist. Geogr..

[10] Bičík, I. *et al*. *Land Use Changes in the Czech Republic 1845–2010. Socio-Economic Driving Forces*. 10.1007/978-3-319-17671-0 (Springer, 2015).

[11] ČSÚ. *Historický lexikon obcí České republiky 1869–2005, 1. a 2. díl (Historical Inventory of Czech Municipalities 1869–2005, vol 1–2). (Český statistický úrad, Prague, 2006)*.

[12] Bar, J. & Franszek, P. *Informator statystyczny do dziejów przemysłu w Galicji: górnictwo i hutnictwo, produkcja, ceny, zbyt, przedsiębiorstwa, zatrudnienie*. (Uniwersytet Jagielloński, 1981).

[13] Lipelt, R. *Informator statystyczny do dziejów społeczno-gospodarczych Galicji. Gospodarka leśna w Galicji w dobie autonomii*. (Towarzystwo Wydawnicze ‘Historia Iagiellonica’, 2017).

[14] Soja, M. *Cykle rozwoju ludności Karpat Polskich w XIX i XX wieku*. (Instytut Geografii i Gospodarki Przestrzennej Uniwersytetu Jagiellońskiego, 2008).

[15] Kladivo P, Ziener K, Roubínek P, Ptáček P (2012). Czech-Polish and Austrian-Slovenian borderland – similarities and differences of development and typology of regions. Morav. Geogr. Reports.

[16] Nowak, K. Przemiany prawno-administracyjne, społeczno-zawodowe i narodowościowe. In *Dzieje Śląska Cieszyńskiego od zarania do czasów współczesnych. Śląsk Cieszyński od Wiosny Ludów do I wojny światowej (1848–1918)* (ed. Panic, I.) 11–20 (Starostwo Powiatowe w Cieszynie, 2013).

[17] Siwek, T. Silesian Identity Across the Internal Border of the EU. In *Borders in Central Europe After the Schengen Agreement* (eds. Tomáš, H., Jaroslav, D. & Milan, J.) 167–176 10.1007/978-3-319-63016-8_10 (Springer, 2018).

[18] Steidl, A. & Stockhammer, E. *Coming and leaving*. *Internal mobility in late Imperial Austria*. (2007).

[19] Zeitlhofer, H., Ehmer, J. & Steidl, A. *Migration Patterns in Late Imperial Austria*. *KMI Working Paper Series* (2006).

[20] Munteanu C (2015). Legacies of 19th century land use shape contemporary forest cover. Glob. Environ. Chang..

[21] Munteanu C (2017). Nineteenth-century land-use legacies affect contemporary land abandonment in the Carpathians. Reg. Environ. Chang..

[22] Konkoly-Gyuró É, Király G, Dezső N, Balázs P, Tirászi Á (2017). Overview of the 18 th-20 th century military surveys in the light of the land cover change assessment in Eastern Central Europe. e-Perimetron.

[23] Alix-Garcia J, Walker S, Radeloff V, Kozak J (2018). Tariffs and trees: The effects of the Austro-Hungarian customs union on specialization and land-use change. J. Econ. Hist..

[24] Ostafin K (2017). Forest cover mask from historical topographic maps based on image processing. Geosci. Data J..

[25] Leyk S, Boesch R, Weibel R (2005). A conceptual framework for uncertainty investigation in map-based land cover change modelling. Trans. GIS.

[26] Lisec A, Navratil G (2014). The Austrian land cadastre: from the earliest beginnings to the modern land information system. Geod. Vestn..

[27] Teibenbacher, P., Kramer, D. & Göderle, W. *An Inventory of Austrian Census**Materials, 1857–1910. Final Report*. **190**, (2012).

[28] *Statistische Übersichten uber die Bevölkerung und den Viehstand von Österreich nach der zählung vom 31. October 1857*. (Kaiserlich-Königlichen Hof- und Staatsdruckerei, 1859).

[29] *Bevölkerung und Viehstand der im Reichsrathe vertretenen Königreiche und Länder dann der Militärgränze nach der Zählung vom 31. December 1869*. (Kaiserlich-Königlichen Hof- und Staatsdruckerei, 1871).

[30] Österreichische Statistik. (Kaiserlich-Königlichen Hof- und Staatsduckerei, 1880).

[31] *Alphabetiſch geordnetes Orts =Verzeichnitz der Kronländer Mähren und Schleſien nach der k. k. politiſchen Adminiſtrations und Gerichtseintheilung entworten von Leopold Raffay*. (Eduard Hötzel, Buchhändler in Olmüz und Neutitſchein, 1850).

[32] *Orts-Repertorium des herzogthums Ober- und Nieder-Schleſien. Auf Grundlage der Volkszählung vom 31. December 1869 bearbeitet von der k. k. Statiſtischen Central-Commiſſion*. (Carl Gerold’s Sohn, 1872).

[33] *Special Orts-Repertorien der im Oesterreichischen Reichsrathe vertretenen Königreiche und Länder. Herausgegeben von der k.k. Statistischen Central-Commission XI. Schlesien*. (K.K. Statistischen Central-Commission. In Commission bei Carl Gerold’s Sohn, 1885).

[34] *Special Orts-Repertorium von Schlesien Neubearbeitung auf grund der ergebnisse der Volkszählung vom 31. December 1890. Herausgegeben von der K. K. Statistischen Central-Commission*. (Alfred Hӧlder K. U. K Hof- und Universitäts-Buchhändler, 1894).

[35] *Gemeindelexikon der im Reichsrate Vertretenen Königreiche und Länder. Bearbeitet auf grund der Ergebnisse der Volkszählung vom 31. Dezember 1900. Herausgegeben von der K. K. Statistischen Zentralkommission. XI. Schlesien*. (K. K. Hof- und Staatsdruckerei, 1906).

[36] *Spezialortsrepertorium der Österreichischen Länder. Bearbeitet auf grund der ergebnisse der Volkszählung vom 31. Dezember 1910. Herausgegeben von der K. K. Statistischen Zentralkommission XI. Schlesien*. (K. K. Hof- und Staatsdruckerei, 1917).

[37] Timár, G. *et al*. *The map sheets of the Second Military Survey and their georeferenced version*. (Arcanum, 2006).

[38] Ostafin K, Kaim D, Siwek T, Miklar A (2020). Harvard Dataverse.

[39] Bičík I, Jeleček L, Štěpánek V (2001). Land-use changes and their social driving forces in Czechia in the 19th and 20th centuries. Land use policy.

[40] Rumpler, H. & Seger, M. *Die Habsburgermonarchie 1848–1918, Band IX/2: Soziale Strukturen. Die Gesellschaft der Habsburgermonarchie im Kartenbild. Verwaltuns-, Sozial-und Infrastrukturen. Nach dem Zensus von 1910*. (VÖAW, 2010).

[41] Burzyński A (1984). Z rozważań nad oceną austriackich powszechnych spisów ludności z lat 1869–1910. Przesz. Demogr. Pol..

