# Supplementary material for: Historical dataset of administrative units with social-economic attributes for Austrian Silesia 1837–1910
Source: Sci Data. 2020 Jun 30;7:208. doi: 10.1038/s41597-020-0546-z (PMC7326999; doi:10.1038/s41597-020-0546-z)
Supplement: Supplementary file 1 — Supplementary Information [file 41597_2020_546_MOESM1_ESM.pdf]

Historical dataset of administrative units with social-economic attributes for Austrian Silesia 1837 – 1910

Full list of administrative divisions
Supplementary Information

Krzysztof Ostafin[1], Dominik Kaim[1], Tadeusz Siwek[2], Anna Miklar[3]

Affiliations
1. Jagiellonian University, Faculty of Geography and Geology, Institute of Geography and Spatial Management
2. University of Ostrava, Faculty of Science, Department of Human Geography and Regional Development
3. Jagiellonian University, Faculty of History, Institute of History
corresponding author: Krzysztof Ostafin (krzysztof.ostafin@uj.edu.pl)

Ostafin, K., Kaim, D., Siwek, T. & Miklar, A. Historical dataset of administrative units with social-economic attributes for Austrian Silesia 1837 – 1910. (2020). https://doi.org/10.7910/DVN/K7YPAF, Harvard Dataverse, V2
https://dataverse.harvard.edu/dataset.xhtml?persistentId=doi:10.7910/DVN/K7YPAF

NATIONAL PROGRAMME
FOR THE DEVELOPMENT OF HUMANITIES

This research was funded by the Ministry of Science and Higher Education, Republic of Poland under the frame of "National Programme for the Development of Humanities" 2015–2020, as a part of the GASID Project (Galicia and Austrian Silesia Interactive Database 1857–1910, 1aH 15 0324 83).

1850

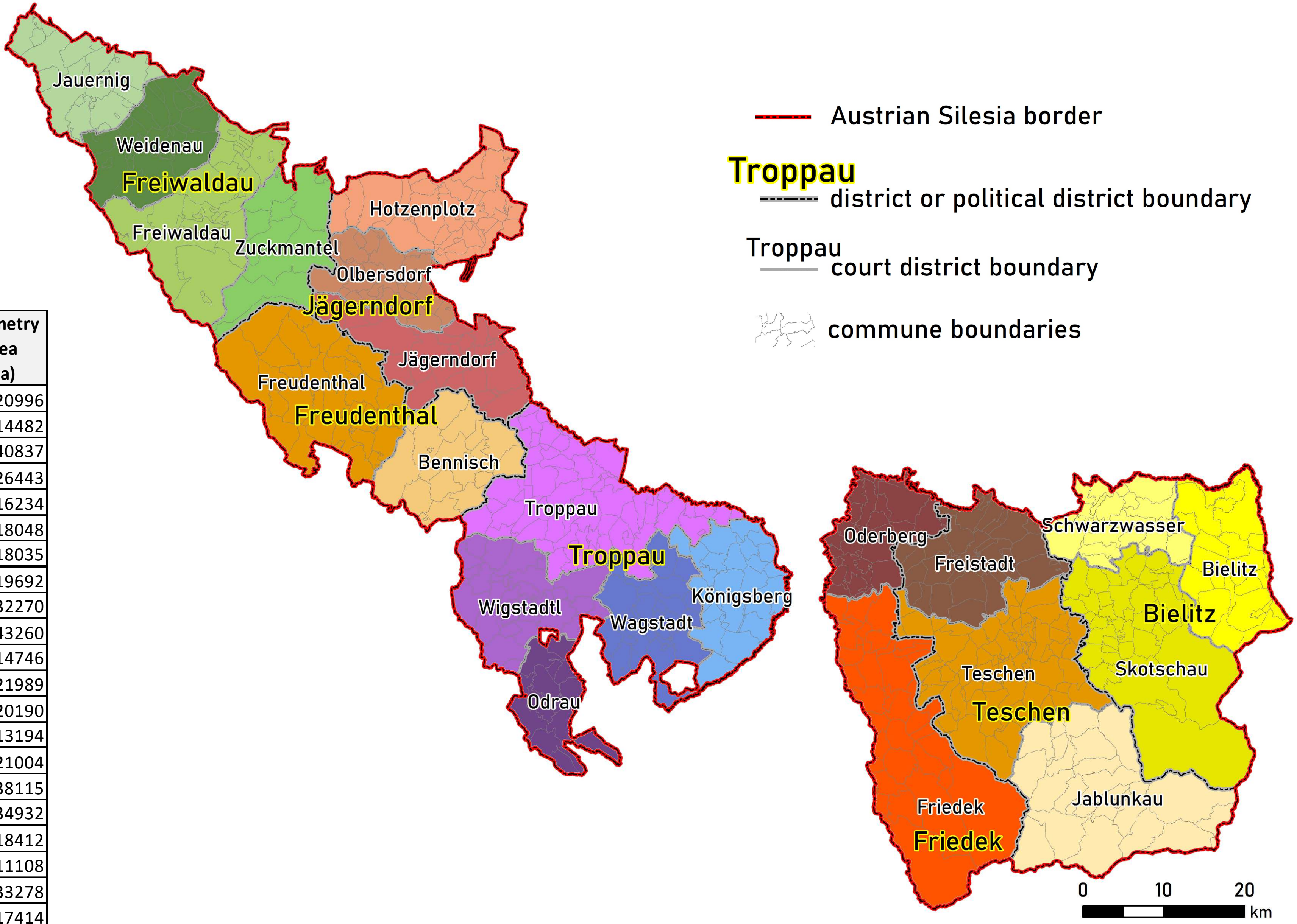

ID	District	ID	Court district	Geometry area (ha)
01	Bielitz	01	Bielitz	20996
		02	Schwarzwasser	14482
		03	Skotschau	40837
02	Freiwaldau	01	Freiwaldau	26443
		02	Jauernig	16234
		03	Weidenau	18048
		04	Zuckmantel	18035
03	Freudenthal	01	Bennisch	19692
		02	Freudenthal	32270
04	Friedek	01	Friedek	43260
		02	Oderberg	14746
05	Jägerndorf	01	Hotzenplotz	21989
		02	Jägerndorf	20190
		03	Olbersdorf	13194
06	Teschen	01	Freistadt	21004
		02	Jablunkau	38115
		03	Teschen	34932
07	Troppau	01	Königsberg	18412
		02	Odrau	11108
		03	Troppau	33278
		04	Wagstadt	17414
		05	Wigstadtl	20234

Districts and court districts division according to Alphabetiſch geordnetes Orts =Verzeichnitz der Kronländer Mähren und Schleſien nach der k. k. politiſchen Adminiſtrations und Gerichtseintheilung entworten von Leopold Raffay. (Verlag von Eduard Hötzel, Buchhändler in Olmüz und Neutitſchein, 1850).

1857

ID	District	Geometry area (ha)	Statistical area (ha)	Difference (%)
00	Troppau Stadt	1090	1093	-0.3
01	Bennisch	19692	19681	0.1
02	Bielitz	20996	21004	0.0
03	Freistadt	21004	21004	0.0
04	Freiwaldau	26443	26471	-0.1
05	Freudenthal	32270	32341	-0.2
06	Friedek	43260	43217	0.1
07	Hotzenplotz	21990	21867	0.6
08	Jablunkau	38115	38095	0.1
09	Jägerndorf	20190	20141	0.2
10	Jauernig	16234	16228	0.0
11	Königsberg	18412	18415	0.0
12	Oderberg	14746	14732	0.1
13	Odrau	11108	11106	0.0
14	Olbersdorf	13194	13178	0.1
15	Schwarzwasser	14482	14502	-0.1
16	Skotschau	40837	40858	-0.1
17	Teschen	34932	34930	0.0
18	Troppau	32188	32168	0.1
19	Wagstadt	17414	17436	-0.1
20	Weidenau	18048	18012	0.2
21	Wigstadtl	20234	20256	-0.1
22	Zuckmantel	18035	18012	0.1

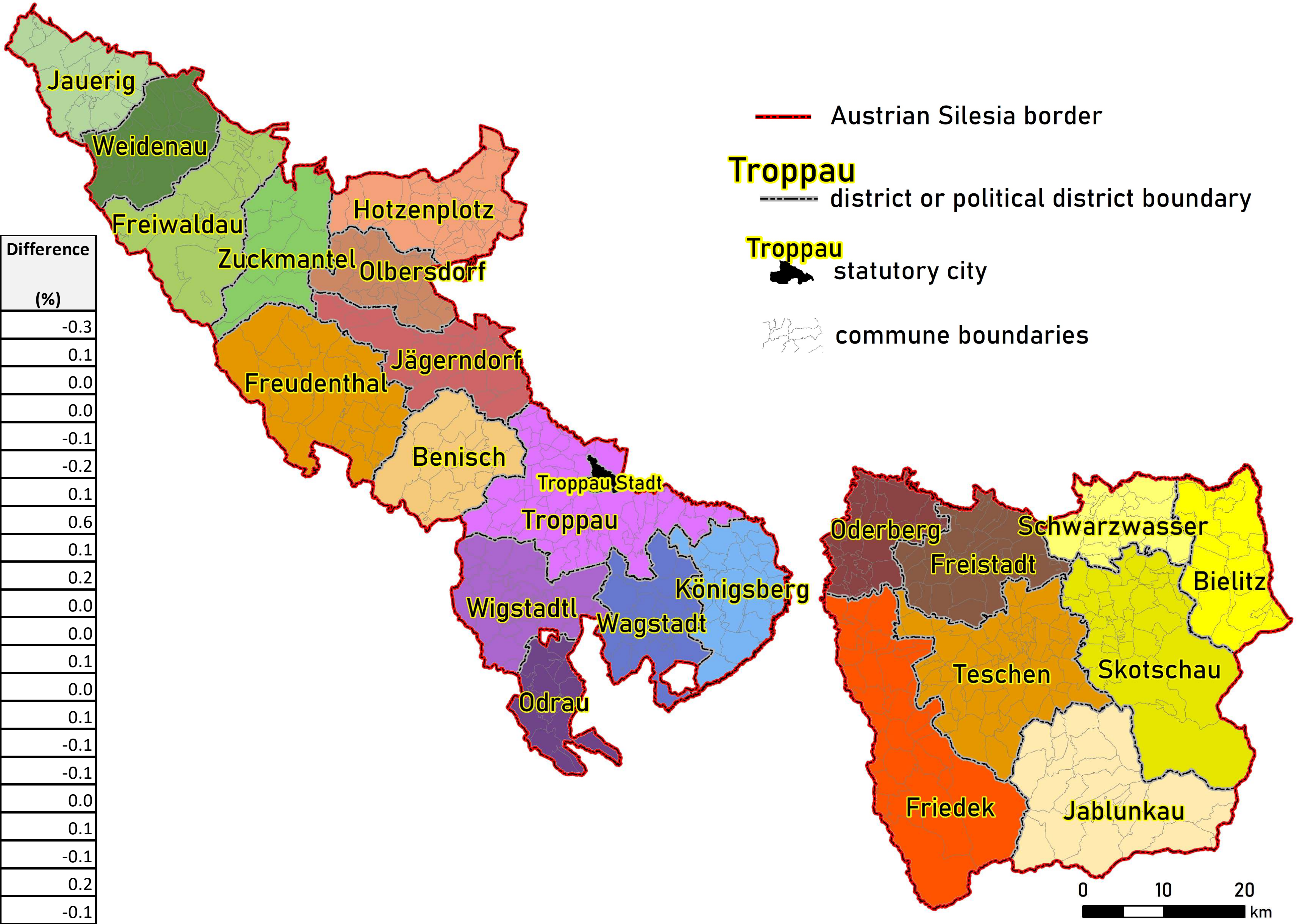

Districts division and statistical area according to Statistische Übersichten uber die Bevölkerung und den Viehstand von Österreich nach der zählung vom 31. October 1857. (Kaiserlich-Königlichen Hof- und Staatsdruckerei, 1859)

1869

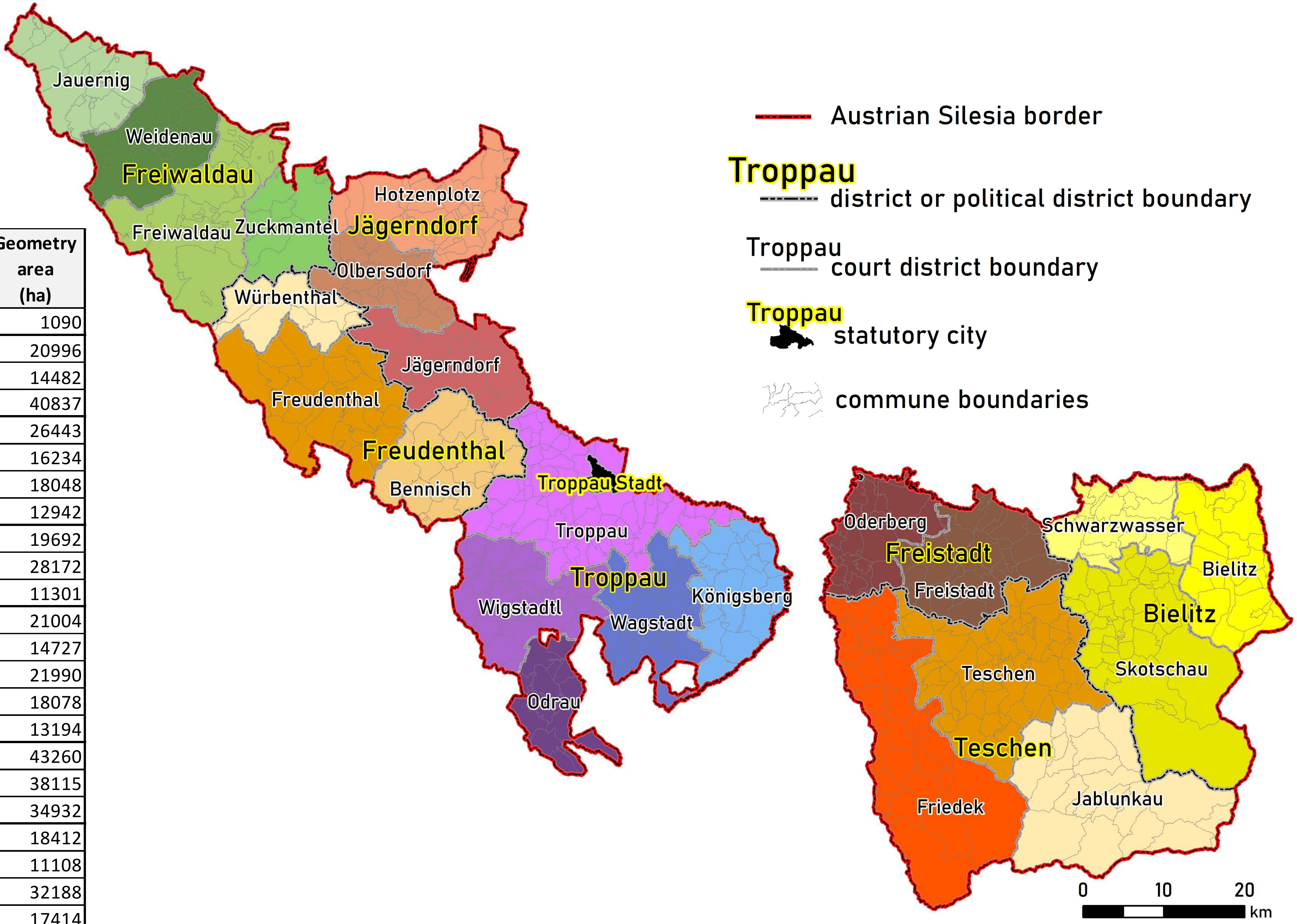

ID	District	ID	Court district	Geometry area (ha)
00	Troppau Stadt	01	Troppau Stadt	1090
01	Bielitz	01	Bielitz	20996
		02	Schwarzwasser	14482
		03	Skotschau	40837
02	Freiwaldau	01	Freiwaldau	26443
		02	Jauernig	16234
		03	Weidenau	18048
		04	Zuckmantel	12942
03	Freudenthal	01	Bennisch	19692
		02	Freudenthal	28172
		03	Würbenthal	11301
04	Freistadt	01	Freistadt	21004
		02	Oderberg	14727
05	Jägerndorf	01	Hotzenplotz	21990
		02	Jägerndorf	18078
		03	Olbersdorf	13194
06	Teschen	01	Friedek	43260
		02	Jablunkau	38115
		03	Teschen	34932
07	Troppau	01	Königsberg	18412
		02	Odrau	11108
		03	Troppau	32188
		04	Wagstadt	17414
		05	Wigstadtl	20234

Districts and court districts division according to Orts-Repertorium des herzogthums Ober- und Nieder-Schleſien. Auf Grundlage der Volkszählung vom 31. December 1869 bearbeitet von der k. k. Statiſtischen Central-Commiſſion. (Carl Gerold’s Sohn, 1872).

1880

ID	District	ID	Court district	Geometry area (ha)	Statistical area (ha)	Difference (%)
00	Statutory city	01	Troppau Stadt	1090	1093	-0.3
		02	Bielitz Stadt	497	497	0.0
		03	Friedek Stadt	1022	1023	-0.1
01	Bielitz	01	Bielitz	20499	20497	0.0
		02	Schwarzwasser	14482	14480	0.0
		03	Skotschau	40837	40847	0.0
02	Freiwaldau	01	Freiwaldau	26443	26454	0.0
		02	Jauernig	16234	16217	0.1
		03	Weidenau	18048	18034	0.1
		04	Zuckmantel	12942	12933	0.1
03	Freudenthal	01	Bennisch	19692	19690	0.0
		02	Freudenthal	28172	28170	0.0
		03	Würbenthal	11301	11302	0.0
04	Freistadt	01	Freistadt	21004	21006	0.0
		02	Oderberg	14727	14635	0.6
05	Jägerndorf	01	Hotzenplotz	12741	12738	0.0
		02	Jägerndorf	18078	18091	-0.1
		03	Olbersdorf	13194	13208	-0.1
		04	Hennersdorf	9249	9230	0.2
06	Teschen	01	Friedek	42239	42209	0.1
		02	Jablunkau	38115	38117	0.0
		03	Teschen	34932	34912	0.1
07	Troppau	01	Königsberg	17711	17710	0.0
		02	Odrau	11108	11110	0.0
		03	Troppau	32889	32872	0.1
		04	Wagstadt	17414	17435	-0.1
		05	Wigstadtl	20234	20220	0.1

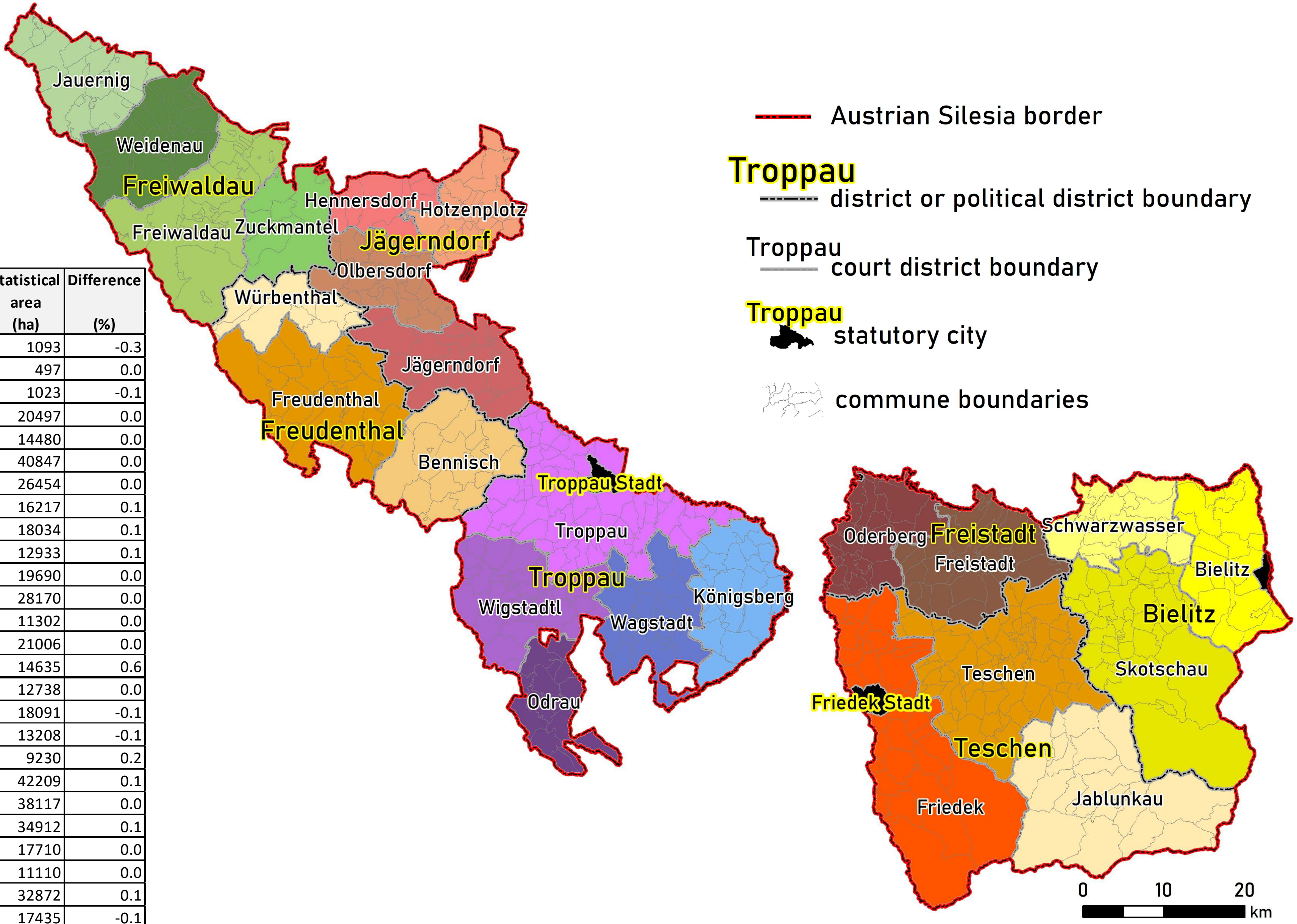

Districts and court districts division according to Special Orts-repertorien der im Oesterreichischen Reichsrathe vertretenen Königreiche und Länder. Herausgegeben von der k.k. Statistischen Central-Commission XI. Schlesien, Wien. (Verlag der K.K. Statistischen Central-Commission. In Commission bei Gerold's Sohn, 1885).

Statistical area according to Statistisches Jahrbuch für das Jahr 1881. Herausgegeben von der k.k. Statistischen Central-Commission, Wien. (Kaiserlich-Königlichen Hof- und Staatsdruckerei, In Commission bei Carl Gerold's Sohn, 1884).

1890

ID	District	ID	Court district	Geometry area (ha)
00	Statutory city	01	Troppau Stadt	1090
		02	Bielitz Stadt	497
		03	Friedek Stadt	1022
01	Bielitz	01	Bielitz	20499
		02	Schwarzwasser	14482
		03	Skotschau	40837
02	Freiwaldau	01	Freiwaldau	26443
		02	Jauernig	16234
		03	Weidenau	18048
		04	Zuckmantel	12942
03	Freudenthal	01	Bennisch	19692
		02	Freudenthal	28172
		03	Würbenthal	11301
04	Freistadt	01	Freistadt	22384
		02	Oderberg	13346
05	Jägerndorf	01	Hotzenplotz	12741
		02	Jägerndorf	18078
		03	Olbersdorf	13194
		04	Hennersdorf	9249
06	Teschen	01	Friedek	42239
		02	Jablunkau	38115
		03	Teschen	34932
07	Troppau	01	Königsberg	17711
		02	Odrau	11108
		03	Troppau	32889
		04	Wagstadt	17414
		05	Wigstadtl	20234

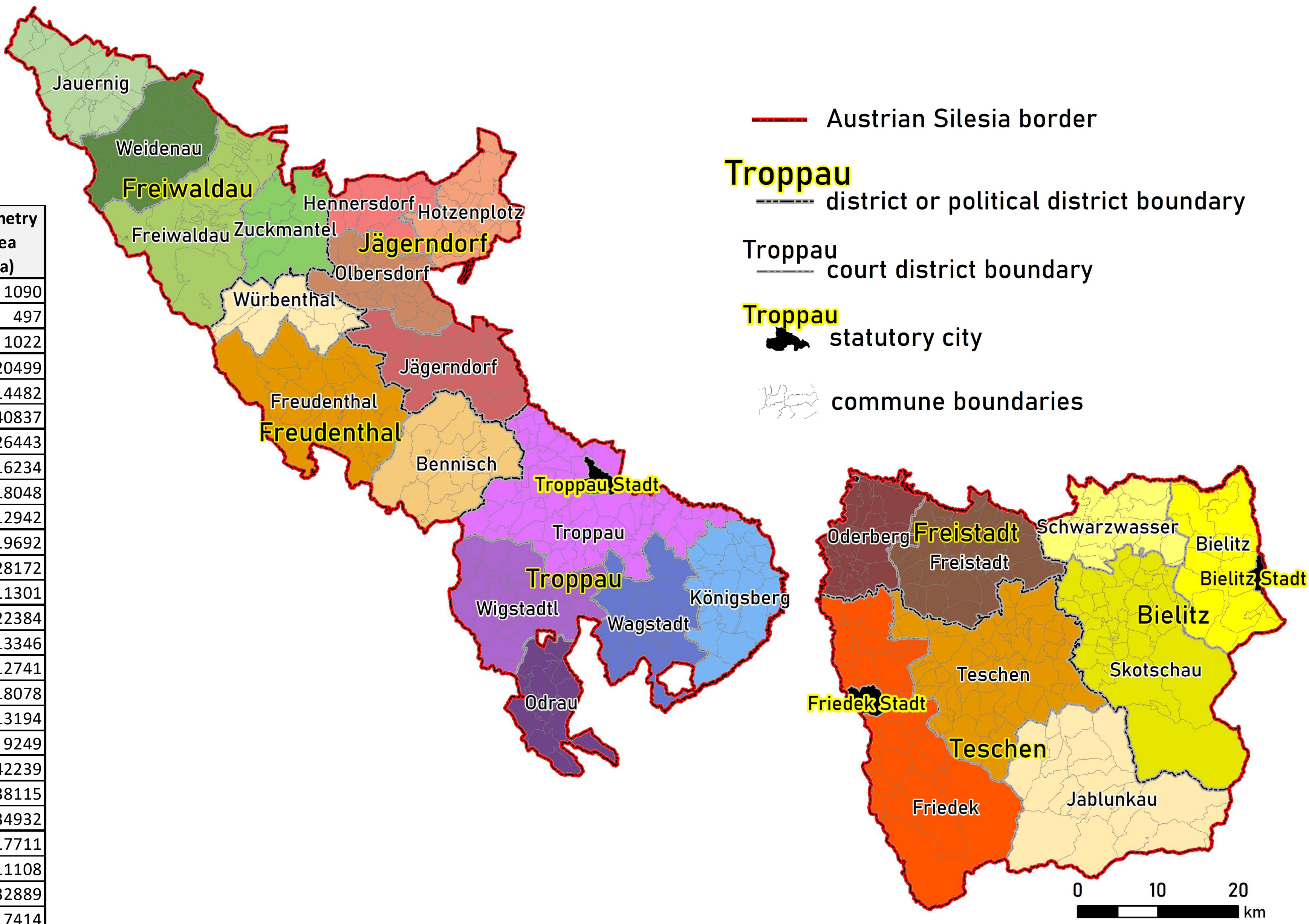

Districts and court districts division according to Special Orts-Repertorium von Schlesien Neubearbeitung auf grund der ergebnisse der Volkszählung vom 31. December 1890. Herausgegeben von der K. K. Statistischen Central-Commission. (1894).

1900

ID	Political district	ID	Court district	Geometry area (ha)	Statistical area (ha)	Difference (%)
00	Statutory city	01	Troppau Stadt	1090	1091	-0.1
		02	Bielitz Stadt	497	497	0.0
		03	Friedek Stadt	1022	1023	-0.1
01	Bielitz	01	Bielitz	20499	20498	0.0
		02	Schwarzwasser	14482	14480	0.0
		03	Skotschau	40837	40836	0.0
02	Freiwaldau	01	Freiwaldau	26443	26454	0.0
		02	Jauernig	16234	16217	0.1
		03	Weidenau	18048	18035	0.1
		04	Zuckmantel	12942	12933	0.1
03	Freudenthal	01	Bennisch	19692	19691	0.0
		02	Freudenthal	28172	28170	0.0
		03	Würbenthal	11301	11302	0.0
04	Freistadt	01	Freistadt	22384	22391	0.0
		02	Oderberg	13346	13250	0.7
05	Jägerndorf	01	Hotzenplotz	12741	12699	0.3
		02	Jägerndorf	18078	18083	0.0
		03	Olbersdorf	13194	13208	-0.1
		04	Hennersdorf	9249	9232	0.2
06	Teschen	01	Friedek	42239	42212	0.1
		02	Jablunkau	38115	38117	0.0
		03	Teschen	34932	34910	0.1
07	Troppau	01	Odrau	11108	11110	0.0
		02	Troppau	32889	32874	0.0
		03	Wigstadtl	20234	20220	0.1
08	Wagstadt	01	Königsberg	15110	15108	0.0
		02	Wagstadt	20015	20036	-0.1

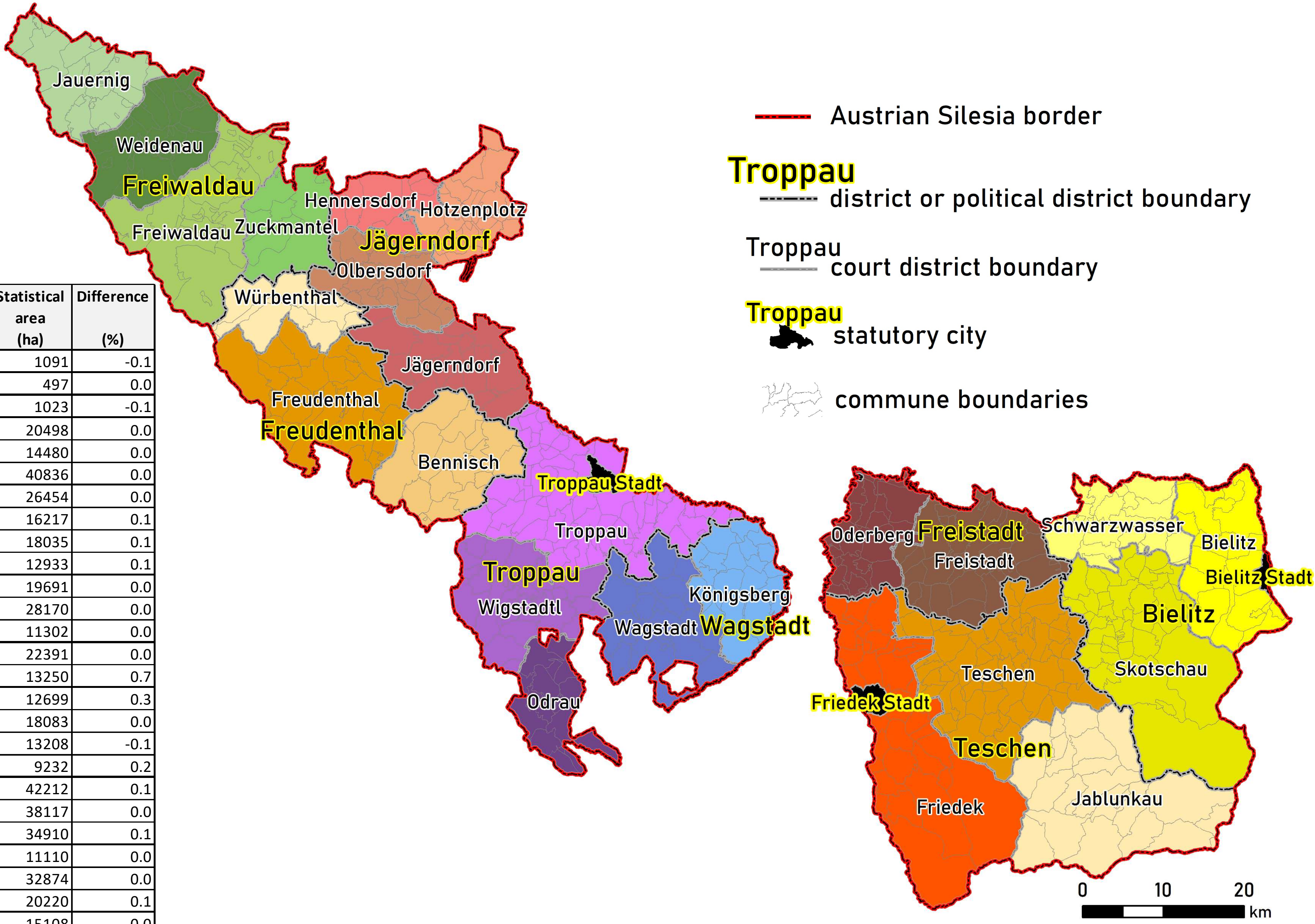

Districts, court districts division and statistical area according to Gemeindelexikon der im Reichsrate Vertretenen Königreiche und Länder. Bearbeitet auf grund der Ergebnisse der Volkszählung vom 31. Dezember 1900. Herausgegeben von der K. K. Statistischen Zentralkommission. XI. Schlesien. (K. K. Hof- und Staatsdruckerei., 1906).

1910

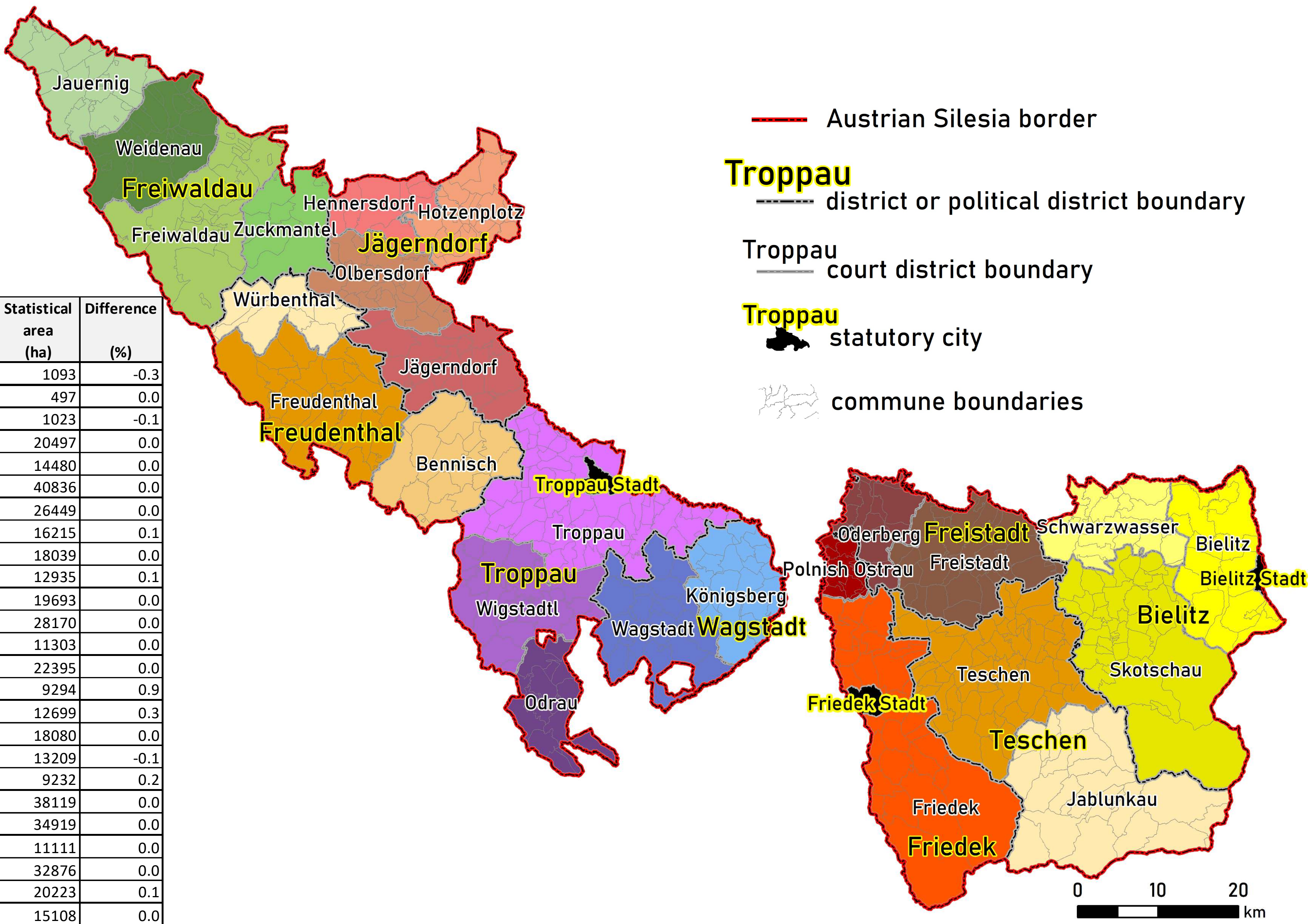

ID	Political district	ID	Court district	Geometry area (ha)	Statistical area (ha)	Difference (%)
00	Statutory city	01	Troppau Stadt	1090	1093	-0.3
		02	Bielitz Stadt	497	497	0.0
		03	Friedek Stadt	1022	1023	-0.1
01	Bielitz	01	Bielitz	20499	20497	0.0
		02	Schwarzwasser	14482	14480	0.0
		03	Skotschau	40837	40836	0.0
02	Freiwaldau	01	Freiwaldau	26443	26449	0.0
		02	Jauernig	16234	16215	0.1
		03	Weidenau	18048	18039	0.0
		04	Zuckmantel	12942	12935	0.1
03	Freudenthal	01	Bennisch	19692	19693	0.0
		02	Freudenthal	28172	28170	0.0
		03	Würbenthal	11301	11303	0.0
04	Freistadt	01	Freistadt	22384	22395	0.0
		02	Oderberg	9382	9294	0.9
05	Jägerndorf	01	Hotzenplotz	12741	12699	0.3
		02	Jägerndorf	18078	18080	0.0
		03	Olbersdorf	13194	13209	-0.1
		04	Hennersdorf	9249	9232	0.2
06	Teschen	01	Jablunkau	38115	38119	0.0
		02	Teschen	34932	34919	0.0
07	Troppau	01	Odrau	11108	11111	0.0
		02	Troppau	32889	32876	0.0
		03	Wigstadtl	20234	20223	0.1
08	Wagstadt	01	Königsberg	15110	15108	0.0
		02	Wagstadt	20015	20036	-0.1
09	Friedek	01	Friedek	42239	42212	0.1
		02	Polnisch Ostrau	3965	3959	0.2

Districts, court districts division and statistical area according to Spezialortsrepertorium der Österreichischen Länder. Bearbeitet auf grund der ergebnisse der Volkszählung vom 31. Dezember 1910. Herausgegeben von der K. K. Statistischen Zentralkommission XI. Schlesien. (Verlag der K. K. Hof- und Staatsdruckerei., 1917).
